# Preparation of Cotton-Wool-Like Poly(lactic acid)-Based Composites Consisting of Core-Shell-Type Fibers

**DOI:** 10.3390/ma8115434

**Published:** 2015-11-24

**Authors:** Jian Wang, Pin Zhou, Akiko Obata, Julian R. Jones, Toshihiro Kasuga

**Affiliations:** 1 Department of Frontier Materials, Nagoya Institute of Technology, Gokiso-cho, Showa-ku, Nagoya 466-8555, Japan; j.wang.671@nitech.jp (J.W.); p.zhou.943@nitech.jp (P.Z.); obata.akiko@nitech.ac.jp (A.O.); 2 Department of Materials, Imperial College London, South Kensington Campus, London SW7 2BP, UK; julian.r.jones@imperial.ac.uk

**Keywords:** biomaterial, composite, poly(lactic-co-glycolic acid), poly(lactic acid), core-shell-type fiber, coaxial electrospinning, vaterite, siloxane, ion release, cotton wool-like material

## Abstract

In previous works, we reported the fabrication of cotton-wool-like composites consisting of siloxane-doped vaterite and poly(l-lactic acid) (SiVPCs). Various irregularly shaped bone voids can be filled with the composite, which effectively supplies calcium and silicate ions, enhancing the bone formation by stimulating the cells. The composites, however, were brittle and showed an initial burst release of ions. In the present work, to improve the mechanical flexibility and ion release, the composite fiber was coated with a soft, thin layer consisting of poly(d,l-lactic-co-glycolic acid) (PLGA). A coaxial electrospinning technique was used to prepare a cotton-wool-like material comprising “core-shell”-type fibers with a diameter of ~12 µm. The fibers, which consisted of SiVPC coated with a ~2-µm-thick PLGA layer, were mechanically flexible; even under a uniaxial compressive load of 1.5 kPa, the cotton-wool-like material did not exhibit fracture of the fibers and, after removing the load, showed a ~60% recovery. In Tris buffer solution, the initial burst release of calcium and silicate ions from the “core-shell”-type fibers was effectively controlled, and the ions were slowly released after one day. Thus, the mechanical flexibility and ion-release behavior of the composites were drastically improved by the thin PLGA coating.

## 1. Introduction

Numerous works on bone regeneration using bioactive materials have been reported. Recently, significant attention has been devoted to bone tissue engineering [1]. Bone-void fillers, which have a connective porous structure, are effective for bone regeneration; various materials, such as hydroxyapatite, β-tricalcium phosphate, and bioactive glass, have been commercialized as fillers [2,3].

Hench *et al*. reviewed that calcium and silicate ions released from bioactive glasses implanted in bone voids have a stimulatory effect on bone regeneration [4,5]. These ions activate osteogenesis and angiogenesis through mediation by insulin-like growth factor (IGF), transforming growth factor β (TGF-β), bone morphogenic proteins (BMPs), vascular endothelial growth factor (VEGF), *etc.* [6]. Xynos *et al*. reported that calcium and silicate ions increase the proliferation of human osteoblasts through gene activation [7,8].

Inorganic-organic composite scaffolds with controllable degradation and bioactive properties are receiving considerable interest for bone tissue regeneration [9,10,11]. In our group, some polymer-ceramic hybrids or composites with the ability to release calcium and silicate ions have been prepared to stimulate the regeneration [12,13,14]. Among them, one of the most promising materials is a composite consisting of poly(l-lactic acid) (PLLA) and siloxane-doped calcium carbonate (vaterite) (SiV) particles, which are derived from aminopropyltriethoxysilane and amorphous calcium carbonates (ACCs) [15]. The siloxane groups on the particle surfaces create amide-I bonds with the carboxy groups at the end of the PLLA chain, and the Ca^2+^ ions in ACC coordinate with the carboxy groups; the SiV particles in the composite are embedded tightly in the PLLA matrix [13]. The SiV-PLLA composite (hereafter denoted by SiVPC) exhibited the ability to release calcium and silicate ions, and enhanced the cell proliferation and differentiation in culture tests using murine osteoblast-like MC3T3-E1 cells [12,13].

Using an electrospinning technique, the SiVPC can be easily shaped into long fibers with diameters ranging from submicrometers to several tens of micrometers. In this process, a kneaded mixture of PLLA and SiV particles at ~200 °C is dissolved in chloroform, while a high voltage source is utilized to inject a charge of a certain polarity, which is accelerated toward a collector of opposite polarity, in the solution. As a result, nonwoven SiVPCs can be obtained [13].

The resulting SiVPC fibrous material containing 20 vol % (30 wt %) of SiV showed hydroxyapatite-forming ability by soaking in simulated body fluid (SBF), while the composite containing 47 vol % (60 wt %) of SiV exhibited the rapid forming ability [14]. The considerable amount of large spaces between the fibers in the nonwoven composite allowed the cell ingrowth and proliferation. An *in vivo* test using New Zealand rabbits showed excellent bone formation around the fibers containing 47 vol % of SiV [13].

To repair irregular bone defects, bone-filling materials with the shapes of granules or porous blocks are used during the healing process. An improvement of their mechanical flexibility would increase the interest in them. Although nonwoven materials are very promising for bone regeneration, their thickness is usually limited to ~0.1–0.2 mm. In our earlier work, cotton-wool-like SiVPCs were developed using a modified electrospinning method [16]. From the viewpoint of the significance for *in vitro* bioactivity and animal tests, cotton-wool-like SiVPCs containing 47 vol % of SiV are expected to be one of the best-performing materials. However, as the fibers, owing to the high SiV content, are brittle, the cotton-wool-like structure may collapse due to compression during handling.

To improve the mechanical brittleness, well-characterized biodegradable poly(lactide-co-glycolide) (PLGA), which is a copolymer of poly(lactic acid) (PLA) and poly(glycolic acid) (PGA), can be used as the matrix polymer in SiVPCs. PLGA is amorphous, as the PLA and PGA polymer chains are not tightly packed [17]. Tensile properties, especially ductility, could be expected to improve using PLGA instead of PLLA as the matrix polymer. Although the composite containing PLGA as the matrix polymer exhibited better mechanical properties than the SiVPC with the PLLA matrix, almost all silicate ions initially dissolved in a burst-release fashion after being soaked in aqueous solution [14]. This may originate from the high hydrophilicity and degradability of PLGA.

We hypothesized that coating a SiVPC fiber containing 47 vol % of SiV with a thin PLGA layer, *i.e.*, preparing a so-called “core-shell”-type structure, may resolve the issues described above; as the thin PLGA layer could prevent the direct contact of the SiVPC with water immediately after soaking the fiber in aqueous solution, the burst release of silicate ions might be inhibited, and the mechanical properties might concurrently improve. The electrospinning method for fabricating core-shell-type structures, *i.e.,* co-electrospinning or coaxial electrospinning, has been mainly applied for the preparation of protein-encapsulated fibers and is reported to be effective for the suppression of the initial burst release of the loaded proteins from the fibers [18].

In the present work, a coaxial electrospinning technique [19] was used to prepare the homogenous PLGA coating layer. The preparation method, mechanical properties, and ion-release behavior of the cotton-wool-like materials are discussed here.

## 2. Results and Discussion

### 2.1. Preparation of Core-Shell-Type Fibers

**Figure 1:**
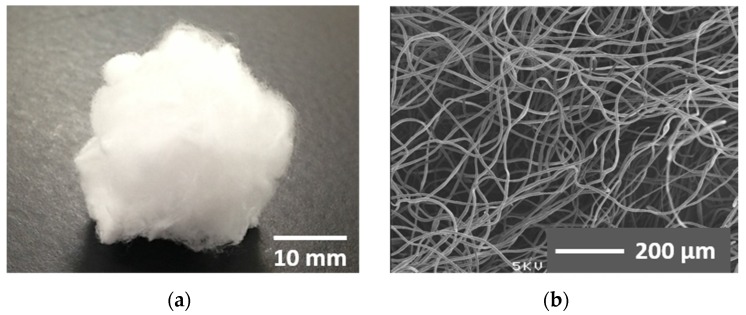
(**a**) Entire view of the cotton-wool-like material prepared using a coaxial electrospinning method; (**b**) SEM image of the material.

The preparation method of cotton-wool-like materials has been reported in our earlier work [16]. After the electrospun fibers fly to rush in ethanol, the electrical charge is neutralized instantly. As a result, no fibers are collected on the ground plate immersed in ethanol. Since almost all of the chloroform is dissolved out into the ethanol, the floating fibers are solidified. In the present study, a coaxial electrospinning method was applied to this technique. Figure 1 shows an entire view and a scanning electron microscopy (SEM) image of the resulting material. A large-sized cotton-wool-like material ~20–30 mm in thickness, not a fiber mat, was obtained Figure 1a. Samples of various sizes can be easily prepared depending on the amount of jetted fibers. Many fibers, which were frizzled and independent of each other, exhibited high porosity and large-sized pores, as seen in Figure 1b.

**Figure 2:**
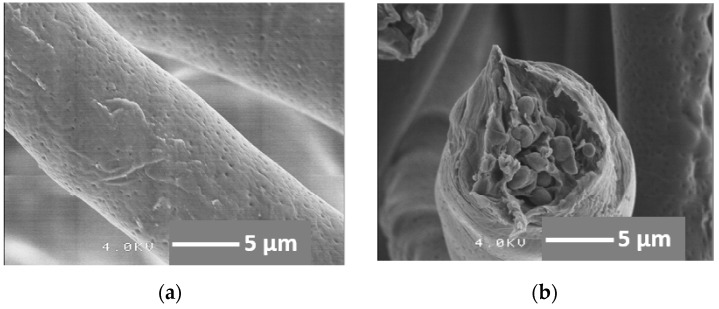
SEM images of the resulting fibrous material fabricated using a coaxial electrospinning method: (**a**) surface of the fiber; (**b**) fracture face after breaking the fiber in liquid nitrogen.

Figure 2 shows the magnified SEM images of a fiber. The fiber diameters were ~10 µm. Many small-sized pits originating from the volatilization of the solvent (chloroform) were observed on the surface, as seen in Figure 2a [20]. In our earlier work, the diameter of the electrospun fibers was reported to be one of the most important parameters for the cell ingrowth [21]. The report showed that mouse osteoblast-like MC3T3-E1 cells on a PLLA fiber mat, with fibers ~10 µm in diameter, could migrate and three-dimensionally grow through the inner fiber gaps. On the other hand, in the case of fiber mats consisting of fibers with a diameter of ~5 µm, the cells grew two-dimensionally, as the gap between the fibers was too small for them to migrate. Therefore, the fiber diameters of the present cotton-wool-like material were controlled to be ~10 µm, although they could be tuned by varying some of the spinning conditions such as the solvent type, viscosity, injecting speed, applied voltage, *etc.*

The cross-sectional view of the fiber fractured in liquid nitrogen shows a core-shell-type structure, as seen in Figure 2b. The core, in which SiV particles in a PLGA matrix can be observed, exhibits a diameter of ~8 µm, while the PLGA coating layer has a thickness of ~2 µm. The fracture face indicates that the SiVPC core fractured catastrophically whereas the PLGA surface layer fractured after necking. Therefore, a thin PLGA layer is successfully coated on the surface of the SiVPC fibers without leaving gaps between them. During the spinning process, the PLGA solution and SiVPC slurry are first ejected through different, coaxial capillary channels—forming a core-shell Taylor cone—and then forced by the electrostatic potential, resulting in the formation of the “core-shell” structured fiber. The advantage of this method is that the core fiber is uniformly coated with a thin layer.

### 2.2. Mechanical Flexibility of Cotton-Wool-Like Materials

**Figure 3:**
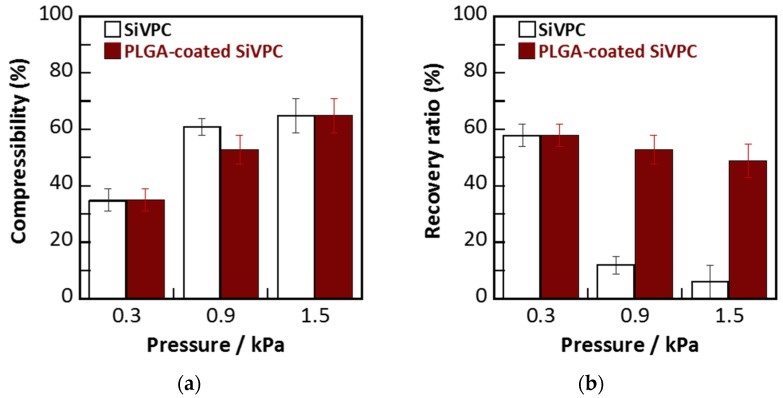
(**a**) Compressibility of cotton-wool-like SiVPC and PLGA-coated SiVPC; (**b**) recovery ratios of the materials. The error bar shows the standard deviation (*n* = 4).

Figure 3 shows the compressibilities and recovery ratios of the cotton-wool-like materials, *i.e.*, SiVPC and PLGA-coated SiVPC consisting of the “core-shell”-type fibers. For all materials, the compressibility increased with the increase of the applied load, reaching ~60% under 1.5 kPa of pressure. In the recovery test, although the samples showed a recovery ratio of 60% after removing 0.3 kPa of low pressure, the SiVPC lost its recovering ability when subjected to a compressive load of 0.9 or 1.5 kPa due to its brittle fracture. On the other hand, the PLGA-coated SiVPC maintained a recovery ratio of ~50%, even under a compressive load of 0.9 or 1.5 kPa.

**Figure 4:**
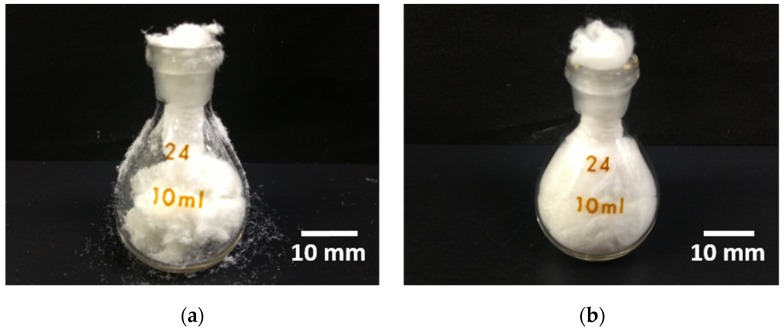
Entire views after cramming 500 g of cotton-wool-like materials into a glass bottle (specific gravity bottle) of 10 mL: (**a**) SiVPC; (**b**) PLGA-coated SiVPC.

Figure 4 shows the handling performance of the cotton-wool-like SiVPC and PLGA-coated SiVPC. For each material, a sample of 500 mg was crammed into a glass bottle (specific gravity bottle) with a volume of 10 mL. The cotton-wool-like SiVPC fibers fractured during cramming and a considerable amount of small-sized broken fibers spilled on the table and outside the bottle; the compression applied to the sample while passing through the narrow bottleneck caused the rupture of several fibers. Owing to the poor recovering ability of the cotton-wool-like SiVPC, some spaces remained unfilled by the fibers. On the other hand, the cotton-wool-like PLGA-coated SiVPC showed an excellent mechanical flexibility, *i.e.*, it was deformed when passing through the narrow bottleneck and managed to completely fill the bottle.

The cotton-wool-like PLGA-coated SiVPC exhibited an excellent handling performance, which may allow this material to play an important role as a bone-void filler.

### 2.3. Dissolution of Calcium and Silicate Ions

The cotton-wool-like SiVPC collapsed into very small-sized pieces after being soaked in Tris buffer solution for three days at 36.5 °C, whereas the PLGA-coated SiVPC maintained its shape even after being soaked for 32 days.

**Figure 5:**
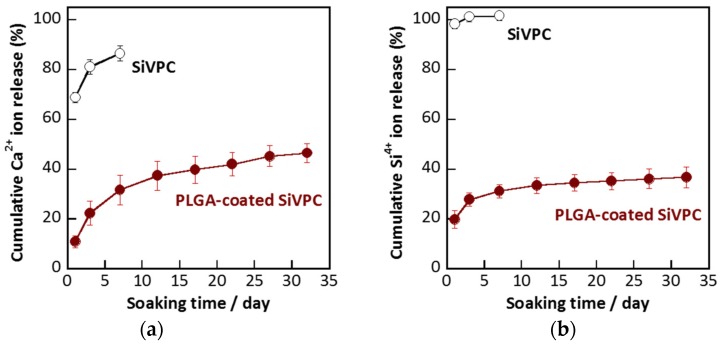
Cumulative ion amounts dissolved from cotton-wool-like materials as a function of the soaking time: (**a**) Ca^2+^ ion; (**b**) silicate ion (here, measured as Si^4+^ ion). The error bar shows the standard deviation (*n* = 3).

Figure 5 shows the cumulative percentages of ions dissolved in the Tris buffer solution from the cotton-wool-like SiVPC and PLGA-coated SiVPC based on the total amounts of the ions in the materials before the soaking. The burst release of the ions from the SiVPC was observed within one day: ~70% of the Ca^2+^ ions and ~98% of the silicate ions in the material were dissolved in the solution. On the other hand, in the case of the PLGA-coated SiVPC, the initial release of the calcium and silicate ions after one day was limited to ~11% and ~20% (*i.e.*, ~20 and ~1.4 ppm), respectively. Subsequently, the ions were continuously released over the considered experimental time span (*i.e.*, 32 days). Thus, the PLGA coating was found to be effective to control the burst release of the ions and achieve their long-term release.

Calcium and silicate ions are important elements in metabolic processes associated with bone formation and calcification [1,2,3,4,5,6,7,8,9]. The control of the ion release is an important factor to enhance the biological capabilities of the composites. In the present work, for the cotton-wool-like SiVPC without coating, almost all the silicate ions and ~70% of the calcium ions were dissolved within one day, *i.e.*, most of the SiV particles were dissolved, leaving a large amount of interconnected pores. As a result, brittle porous fibers composed of a thin PLLA skeleton were formed.

On the other hand, by coating the SiVPC with a PLGA layer, the amount of released ions was successfully controlled. From the morphology of the resulting fibers, as shown in Figure 2a, all fibers were homogenously coated with a PLGA layer, which included small-sized pits due to the volatilization of the solvent (chloroform) during the electrospinning process. PLGA is more hydrophilic than PLLA and more likely to absorb water to swell [22,23]. The good hydrophilicity and high degradation ability might allow the solution to permeate the fibers, especially through the pits. As a result, the ions could dissolve in the solution. Subsequently, owing to the swelling of PLGA in the solution, the permeation route would be narrowed or closed, enabling the control of the ion release and resulting in a long-term and slow delivery. Similar results have been reported for protein-loaded fibers; the initial burst release from the core-shell-type fiber structure is significantly lower, and their release profile is more sustained than that of the protein-blend electrospun fibers. This is due to the core-shell-type fiber structure, which provides a protein reservoir system with a barrier membrane that controls the protein diffusion rate [18,24]. The release profiles can vary by changing the polymer composition used for the shell material; the difference in their pore formation ability can actively contribute to control and optimize the release profile for a variety of applications.

The core-shell-type fiber structure fabricated in the present work appears to be an excellent ion-release control system. The thickness of the coating layer is one of the most important factors for adjusting the ion-release behavior of the materials. Further investigations on the control of the layer thickness are currently in progress. Compared with other methods such as dip-coating, coaxial electrospinning is expected to provide significant advantages in the fabrication of ideal core-shell-type fiber structures, as it can definitely form gaps between the fibers as well as provide pores on the fiber surfaces; besides, it is a one-step preparation method. Additionally, this technique is useful to fabricate fibers of materials that do not typically form fibers via a conventional electrospinning method [25]. The present work would contribute to increasing the amount of SiV in future core composites.

The cotton-wool-like “SiVPC-core/PLGA-shell“-type material, which has several advantages such as a highly porous structure (beneficial for cell migration), remarkable mechanical properties (convenient for filling irregular bone defects), and ion-releasing ability (effective for enhancing cellular activities), is expected to exhibit excellent performances when used as a bone-void filler. Cell culture tests for clarifying these advantages are now in progress.

## 3. Experimental Section

As degradable polymers for fibrous materials, PLLA (LACEA, Mitsui Chemicals, Co., Ltd., Tokyo, Japan; Mw: 140 kDa) and poly(l,d-lactide-co-glycolide) (PLGA, lactide:glycolide = 75:25; Purasorb^®^; Corbion Purac Biomaterials, Amsterdam, The Netherlands; Mw: 195 kDa) were selected. The SiV particles, which were supplied from Yabashi Industries (Ogaki, Japan), were prepared by a carbonation process with methanol, as described in our previous reports [12,13]. The particles exhibited spherical shapes ~1.4 μm in diameter, and their Si content was estimated to be 2.6 wt % by X-ray fluorescence analysis.

PLLA and SiV particles were kneaded mechanically at 200 °C for 10 min to prepare the SiVPC. The ratio of PLLA:SiV was 53:47 in vol % (*i.e.*, 40:60 in wt %). The resulting composites were dissolved in chloroform (Wako Pure Chemical Industries, Osaka, Japan) to prepare an 8 wt % solution of PLLA in SiVPC. PLGA was also dissolved in chloroform to prepare a 15 wt % solution.

**Figure 6:**
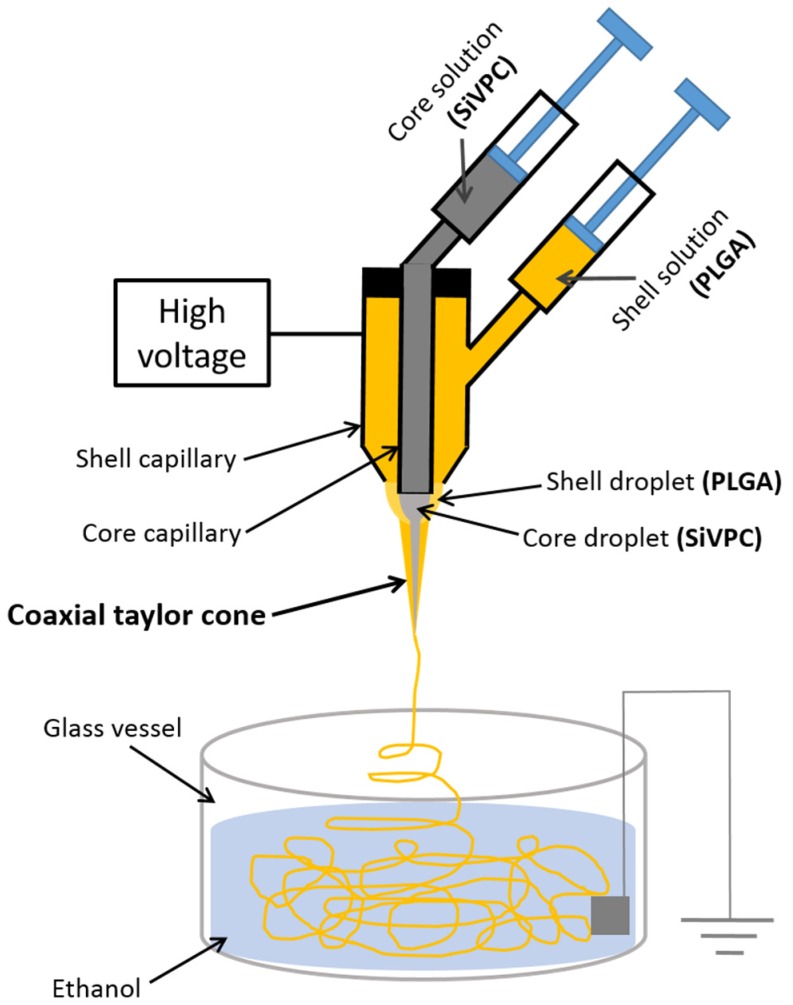
Schematic drawing of coaxial electrospinning system for preparing cotton-wool-like materials.

Figure 6 shows a schematic drawing of the coaxial electrospinning system used in the present work: a concentric metallic nozzle was used for the spinning. A jet was generated on the tip of the droplet, and a core-shell-type fiber was formed after adjusting some parameters such as the applied electric field strength, the viscosity and flow rate of each solution, *etc.* [26,27,28]. In the present work, during the electrospinning, a voltage of 10 kV was applied to the concentric nozzle at room temperature. Each solution was loaded into a syringe pump (FP-W-100, Melquest, Toyama, Japan); the inner diameters of the core-side (*i.e.*, SiVPC) and shell-side (*i.e.*, PLGA) nozzles were 0.5 and 1.10 mm, respectively. The flow rates of the core-side and shell-side solutions were 37.6 and 3.3 µL/min, respectively.

A ground plate was soaked in ethanol and then placed in the vessel (100 mm in diameter). The distance between the concentric nozzle and the vessel used as the collector was 150 mm. A high-tension field was applied to the nozzle. The impressed solution with a core-shell-type structure flowed to the ground plate immersed in the ethanol, while chloroform dissolved. As a result, the solution solidified.

The resulting core-shell-type fibers in ethanol were collected, washed with fresh ethanol, and subsequently dried at room temperature, resulting in the formation of cotton-wool-like materials.

Meanwhile, as a control group, cotton-wool-like SiVPCs without PLGA coating were prepared by electrospinning a 12 wt % PLLA solution containing SiV in chloroform (applied voltage: 15 kV) into the ethanol bath collector described above using an 18 gauge needle.

The resulting fibers were observed by SEM (JSM-6301F, JEOL, Akishima, Japan).

To evaluate the mechanical flexibility of the materials, their compression recovery abilities were examined by following a modified JIS L1097 method. In the tests, 50 mg of each sample was placed in a glass bottle with an inner diameter of 15 mm and a height of 20 mm; then, a cover glass was placed on the sample. The height of the sample, *h_1_*, was measured, and subsequently a weight of 5.4, 16.2, or 27.0 g was placed on the sample for 30 s to obtain an applied load of 0.3, 0.9, or 1.5 kPa, respectively. The height of the sample, *h_2_*, was measured. After removing the weight, the height of the sample, *h_3_*, was measured again 30 s after the removing. The compressibility (%), *Z_1_*, and recovery ratio (%), *Z_2_*, were estimated from the following equations:

*Z_1_* = {(*h_1_* − *h_2_*)/*h_1_*} × 100 (1)

*Z_2_* = {(*h_3_* − *h_2_*)/(*h_1_* − *h_2_*)} × 100 (2)

The ion releasability of the core-shell-type fibrous materials was examined using a Tris buffer solution (pH = 7.4 at 36.5 °C). For the test, 20 mg of the sample was soaked in 10 mL of the solution at 36.5 °C for 32 days. At each time point, the sample was removed from the solution. The amounts of calcium and silicate ions dissolved in the solution were measured by inductively coupled plasma-atomic emission spectroscopy (ICPS-7000, Shimadzu, Kyoto, Japan) (*n* = 3).

## 4. Conclusions

Using a coaxial electrospinning technique, a PLGA thin layer with a thickness of ~2 µm was successfully coated on the surface of SiVPC fibers with a diameter of ~8 µm, obtaining a cotton-wool-like structure. The SiVPC fibers without the coating were considerably brittle and tended to easily break into small-sized fiber pieces under compression; conversely, the PLGA-coated SiVPC fibers were mechanically flexible and maintained their shape even when subjected to a compressive load of 1.5 kPa. The mechanical flexibility of the cotton-wool-like SiVPC was drastically improved by the thin PLGA coating. In addition, the coating effectively controlled the initial burst release of calcium and silicate ions from the SiVPC fibers in Tris buffer solution. The SiVPC fibers collapsed in the solution within three days, whereas the PLGA-coated SiVPC fibers maintained their structure even after being soaked in the solution for one month.

## References

[1] Hench L.L., Polak J.M. (2002). Third generation biomedical materials. Science.

[2] Pietrzak W.S., Ronk R. (2000). Calcium sulfate bone void filler: A review and a look ahead. J. Craniofacial Surg..

[3] Kenny S.M., Buggy M. (2003). Bone cements and fillers: A review. J. Mater. Sci. Mater. Med..

[4] Hench L.L., Xynos I.D., Polak J.M. (2004). Bioactive glasses for *in situ* tissue regeneration. J. Biomater. Sci. Polym. Ed..

[5] Hench L.L., Day D.E., Höland W., Rheinberger V.M. (2010). Glass and medicine. Int. J. Appl. Glass Sci..

[6] Hoppe A., Güldal N.S., Boccaccini A.R. (2011). A review of the biological response to ionic dissolution products from bioactive glasses and glass-ceramics. Biomaterials.

[7] Xynos I.D., Edgar A.J., Buttery L.D.K., Hench L.L., Polak J.M. (2000). Ionic products of bioactive glass dissolution increase proliferation of human osteoblasts and induce insulin-like growth factor II mRNA expression and protein synthesis. Biochem. Biophys. Res. Commun..

[8] Xynos I.D., Edgar A.J., Buttery L.D.K., Hench L.L., Polak J.M. (2001). Gene-expression profiling of human osteoblasts following treatment with the ionic products of Bioglass^®^ 45S5 dissolution. J. Biomed. Mater. Res..

[9] Jones J.R. (2013). Review of bioactive glass: From hench to hybrids. Acta Biomater..

[10] Lu W., Ji K., Kirkham J., Yan Y., Boccaccini A.R., Kellett M., Jin Y., Yang X. (2014). Bone tissue engineering by using a combination of polymer/Bioglass composites with human adipose-derived stem cells. Cell Tissue Res..

[11] Boccaccini A.R., Erol M., Stark W.J., Mohn D., Hong Z., Mano J.F. (2010). Polymer/bioactive glass nanocomposites for biomedical applications: A review. Compos. Sci. Technol..

[12] Obata A., Tokuda S., Kasuga T. (2009). Enhanced *in vitro* cell activity on silicon-doped vaterite/poly(lactic acid) composites. Acta Biomater..

[13] Obata A., Hotta T., Wakita T., Ota Y., Kasuga T. (2010). Electrospun microfiber meshes of silicon-doped vaterite/poly(lactic acid) hybrid for guided bone regeneration. Acta Biomater..

[14] Fujikura K., Obata A., Lin S., Jones J.R., Law R.V., Kasuga T. (2012). Preparation of electrospun poly(lactic acid)-based hybrids containing siloxane-doped vaterite particles for bone regeneration. J. Biomater. Sci. Ploym. Ed..

[15] Nakamura J., Poologasundarampillai G., Jones J.R., Kasuga T. (2013). Tracking the formation of vaterite particles containing aminopropyl-functionalized silsesquioxane and their structure for bone regenerative medicine. J. Mater. Chem. B.

[16] Kasuga T., Obata A., Maeda H., Ota Y., Yao X.F., Oribe K. (2012). Siloxane-poly(lactic acid)-vaterite composites with 3D cotton-like structure. J. Mater. Sci. Mater. Med..

[17] Middleton J.C., Tipton A.J. (2000). Synthetic biodegradable polymers as orthopedic devices. Biomaterials.

[18] Ji W., Sun Y., Yang F., van den Beucken J.J.P., Fan M., Chen Z., Jansen J. (2011). Bioactive Electrospun Scaffolds Delivering Growth Factors and Genes for Tissue Engineering Applications. Pharm. Res..

[19] Su Y., Li X.Q., Wang H.S., He C.L., Mo X.M. (2009). Fabrication and characterization of biodegradable nanofibrous mats by mix and coaxial electrospinning. J. Mater. Sci. Mater. Med..

[20] Huang Z.M., Zhang Y.Z., Kotakic M., Ramakrishna S. (2003). A review on polymer nanofibers by electrospinning and their applications in nanocomposites. Compos. Sci. Technol..

[21] Fujikura K., Obata A., Kasuga T. (2012). Cellular migration to electrospun poly(lactic acid) fibermats. J. Biomater. Sci. Polym. Ed..

[22] Makadia H.K., Siegel S.J. (2011). Poly lactic-co-glycolic acid (PLGA) as biodegradable controlled drug delivery carrier. Polymers.

[23] Zong X., Ran S., Kim K.-S., Fang D., Hsiao B.S., Chu B. (2003). Structure and morphology changes during *in vitro* degradation of electrospun poly(glycolide-co-lactide) nanofiber membrane. Biomacromolecules.

[24] Zhang Y.Z., Wang X., Feng Y., Li J., Lim C.T., Ramakrishna S. (2006). Coaxial Electrospinning of (Fluorescein Isothiocyanate-Conjugated Bovine Serum Albumin)-Encapsulated Poly(ε-caprolactone) Nanofibers for Sustained Release. Biomacromolecules.

[25] Sun Z., Zussman E., Yarin A.L., Wendorff J.H., Greiner A. (2003). Compound Core-Shell Polymer Nanofibers by Co-Electrospinning. Adv. Mater..

[26] Loscertales I.G., Barrero A., Marquez M., Spretz R., Velarde-Ortiz R., Larsen G. (2004). Electrically forced coaxial nanojets for one-step hollow nanofiber design. J. Am. Chem. Soc..

[27] Li D., Xia Y.N. (2004). Direct fabrication of composite and ceramic hollow nanofibers by electrospinning. Nano Lett..

[28] Díaz J.E., Barrero A., Márquez M., Loscertales I.G. (2006). Controlled encapsulation of hydrophobic liquids in hydrophilic polymer nanofibers by co-electrospinning. Adv. Funct. Mater..

